# Testing Failure Mode and Effect Analysis: A Descriptive Empirical Study in Paediatric and Adolescent Hospital Care

**DOI:** 10.1111/jep.70722

**Published:** 2026-11-15

**Authors:** Aino Färlin‐Helin, Noora Narsakka, Sakari Suominen, Outi Tuominen

**Affiliations:** ^1^ Department of Public Health University of Turku Turku Finland; ^2^ University of Eastern Finland Kuopio Finland; ^3^ Department of Nursing Science University of Eastern Finland Turku Finland; ^4^ Southwest Finland Wellbeing Services County Turku Finland; ^5^ University of Skövde Skövde Sweden

**Keywords:** failure mode and effect analysis, hospital pediatric, patient safety, risk assessment, risk management

## Abstract

**Rationale:**

Patient safety incidents may have serious consequences for hospitalised paediatric patients. It is estimated that half of all incidents in paediatric care could be prevented. Proactive risk management methods aim to identify, assess and mitigate potential risks before they result in harm to patients.

**Aims and Objectives:**

To test and introduce the Failure Mode and Effect Analysis method in various processes in paediatric and adolescent department with action research methodology.

**Method:**

Failure Mode and Effect Analysis (FMEA) produces a risk priority number (RPN), calculated by multiplying the values of occurrence, severity and detectability. In this study, FMEA was tested by professionals working in the department with the support of a team leader. A cyclical action research process was used, including the phases of planning, acting, analysis and drawing conclusions. The study was conducted between February and September 2024, conducting one action research cycle.

**Results:**

Multidisciplinary teams (team sizes varied from 3 to 6) from a paediatric department tested the Failure Mode and Effect Analysis method and assessed 8 processes, identifying 59 failure modes (average of 6.75 per process). The RPN range was from 2 to 36. A total of 52 corrective actions were developed targeting high‐risk failure modes. Actions ranged from simple (e.g., information sharing) to complex (e.g., equipment changes). FMEA enabled the teams to proactively prioritise and address risks.

**Conclusion:**

The Failure Mode and Effect Analysis is a suitable method for assessing various processes in a paediatric hospital. Action research was an appropriate method to support the testing of FMEA and to introduce a proactive risk management method to study participants.

## 1 Introduction

Risk management in healthcare aims to prevent patient safety incidents in order to provide safe and high‐quality healthcare services. Incidents can be near‐miss events such as specimen handling errors in operation rooms [1], delays in care [2] or events that occurred to the patient [3]. The most serious consequences to the patient vary from severe harm for example causing disabilities [3] or even patient's death [4]. Learning from incidents is still difficult, due to professional politics of incident classification, confidentiality policies, the enhancement of organisation reporting processes and the focus on incidents with serious consequences [5]. It is estimated that half of the adverse event (AE) incidents could be preventable [6, 7], for example errors in medication processes or patient identification [8]. Patient safety incidents increase the costs of care [3, 9]. There are variations in the organisations’ incident investigations and follow‐up processes.

The common types of adverse events (AEs) in neonatal and paediatric hospital care are healthcare associated infections [10, 11], harm to skin, tissue or blood vessels [2, 12], medication errors [8, 13, 14] and the use of medical devices [8]. The identification of AEs is based on the views of the person who is the reporter of the event, they are mainly reported by nursing staff [15, 16]. Low error reporting rates of AEs have been identified [14]. Perceived barriers to reporting include fear of personal consequences, feeling guilty for potential harm and lack of time [16]. An individual AE may be investigated in depth, for example, with a root cause analysis. This method is used but seldom reported [15]. Reactive risk management in hospitals refers to the strategies and actions taken to manage risks and AEs after they have occurred or been identified [17]. Incident reporting and investigation are commonly used in health care [18, 19] and reporting systems vary between countries.

Proactive risk management in a hospital environment involves identifying, assessing and mitigating potential risks before they result in harm or AEs. Proactive risk management tools have been implemented in health care from other high safety industries. Failure mode and effect analysis (FMEA) is one of the proactive risk management tools for error reduction, quality improvement [20] and gathering data for decision‐making [21]. The areas where FMEA has been applied in healthcare have been divided into healthcare processes, hospital management, hospital informatisation and medical equipment or production [20]. FMEA has been used in paediatric hospital care settings, for example to assess the medication processes [22, 23], postoperative discharge delays [24] and safe early mobility processes in Paediatric Intensive Care Units [25]. Furthermore, FMEA has been utilised for complex new care processes such as implementing iodine‐131 meta‐iodobenzylguanidine (MIBG) therapy in the treatment of children diagnosed with high‐risk neuroblastoma [26]. Using a multi‐professional team [26, 27] with 3‐8 members [28] is crucial to the identification of potential risks. However, using FMEA requires familiarity and time [29, 30], which can be a challenge due to staff shortages [29].

In FMEA, the Risk Priority Number (RPN) helps to prioritise corrective actions. RPN is calculated by multiplying severity (S), occurrence (O) and detection (D) [31]. Additionally, the FMEA has been used with revised scores [32, 33, 34, 35] due to different guidelines [36] or for making scales suitable for specific institutions [37]. The FMEA often identifies multiple risks. High‐risk RPNs have been found in several processes in paediatric care hospitals. In drug administration process high‐risk RPNs were related to drug calculation errors [22, 35], wrong dilution [23, 34] and wrong rate [23]. Evaluated processes included actions to improve the medication process, such as double‐checking and signing for each medication prescription and using the same references for standard doses and dilutions [23]. However, studies using FMEA usually recommend corrective actions, but seldom report a reduction in failures. Methods for evaluating the effectiveness of proposed corrective actions are rarely reported [29]. Despite the growing use of FMEA in healthcare, there is limited evidence on its application across multiple clinical processes within paediatric specialised healthcare, particularly in the Finnish hospital context. In addition, few studies have explored FMEA implementation using an action research approach in real‐world clinical environments. This study addresses these gaps by providing empirical evidence on the use of FMEA across multiple clinical processes in a real‐world paediatric hospital setting and by demonstrating its application through an action research approach.

The aim of this study was to apply FMEA in a Finnish paediatric and adolescent hospital department using an action research approach and to examine its applicability as a proactive patient safety method in paediatric specialised healthcare.

## 2 Methods

### 2.1 Methodology

The study was conducted using the methodology of action research. Action research is focused in acting and conducting research simultaneously by the collaboration of researchers and practitioners [38]. In health care research, the approach has been increasingly used in order to implement changes and to develop health service delivery. In hospital settings, it has been previously used, for example, to improve care processes, clinical practice, quality of care, staff collaboration [39], and patient safety [40]. Several studies have applied action research in the context of paediatric care to implement and test tools [41]. Therefore, action research as a methodology was selected to test and introduce the FMEA method to the use of healthcare workers in a paediatric and adolescent department.

Action research is conducted as a cyclical process [42]. In the present study, one cycle was conducted, including the phases of planning, acting, analysis and conclusions. In the phase of planning, plans for the research process were set up and collaborative work with the professionals involved initiated. The first author gathered information concerning the FMEA method and planned preliminary procedures for the subsequent phases. Planning for the research process was further refined in co‐operation with the stakeholders of the participating department. To proceed to the next phase, the FMEA method was introduced to nurse managers and physicians. To facilitate the process, the first author attended the nurse manager's weekly meetings and visited units to inform the staff about the ongoing study.

In the phase of acting, the risk assessment according to the FMEA method (Figure 1), was carried out facilitated by the first author and the professionals working in the department. Data collected during the risk assessment included FMEA assessment. Furthermore, feedback was collected from the members of the risk assessment teams after conducting the assessments. In the phase of analysis, the data collected during the FMEA assessment processes were analysed to draw conclusions about using the FMEA method in the context of paediatric and adolescent care. The FMEA template was used for data collection. In the phase of conclusion, conclusions were drawn based on the analysis carried out and the feedback collected from the professionals participating in the FMEA assessments.

**Figure 1:**
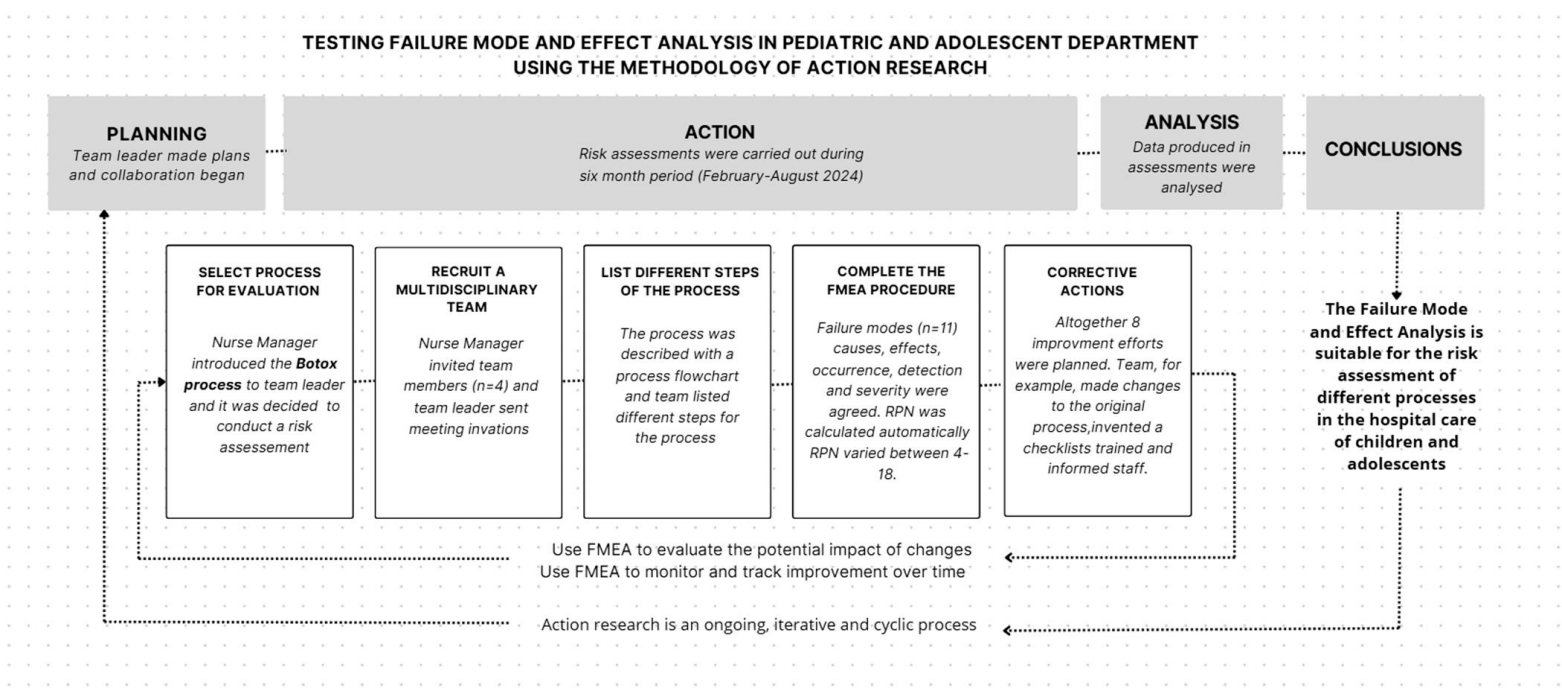
Testing failure mode and effect analysis in paediatric and adolescent hospital department. An example of the Botox process (Table 1).

In the research process, the first author had an insider role working in a professional capacity in the department. During the assessments she acted as a team leader, facilitating the process and guiding the assessment teams. For example, the team leader tried to guide and advance the assessments by posing questions from various perspectives. Having an insider role facilitated familiarity and trust with the professionals collaborating in the study and confirmed pre‐understanding on the study topic, the organizational processes and the policies in the participating organisation [43], this resulted in time saving in the research process. In contrast, the researcher was part of the work culture, possibly limiting her objectivity when conducting the study. To minimise bias, a researcher diary was used to reflect on the first author's role in the project [44]. Furthermore, for transparency, the first author's role when conducting the assessments has been reported in detail.

This study is reported in accordance with the SQUIRE 2.0 (Revised Standards for Quality Improvement Reporting Excellence) guidelines. No Artificial Intelligence Generated Content (AIGC) tools were used in the development of this manuscript.

### 2.2 Setting and Participants

This study was conducted in a university hospital Department of Paediatrics and Adolescent Medicine in Finland that provides tertiary‐level specialist healthcare for children and adolescents. The Department provides a broad range of specialised paediatric services, including care for critically ill children, neonates and children and adolescents requiring medical, surgical, neurological and oncological care. The department is divided into six divisions: paediatric and adolescent medicine, paediatric intensive care unit (PICU), neonatology, paediatric neurology, paediatric surgery, paediatric and adolescent haematology and oncology. The department is responsible for providing specialist health services for children under 16 years old for a population of a corresponding age of 158,000 in the region. The department comprises 70 hospital beds, divided into the following wards neonatal intensive care unit (*n* = 22), paediatric intensive care unit (*n* = 6), paediatric surgical ward (*n* = 14), paediatric ward (*n* = 19) and paediatric haematology ward (*n* = 9), where *n* indicates the number of beds, persons or risk assessments, as applicable. The number of inpatients in 2024 was 3663 (care days 15.764) and the number of outpatients was 24.625 (outpatient care appointments 86.019). The staff consisted of physicians (*n* = 57), nursing staff (*n* = 307) and others (*n* = 46), for example physiotherapists and psychologists, were eligible to participate in the study. In the Department of Paediatric and Adolescent Medicine, reactive risk management through incident reporting is used to systematically assess and ensure patient safety and quality. Proactive risk management methods have not been introduced before.

### 2.3 Description of the Failure Mode and Effect Analysis Method Process

The FMEA process consists of the following steps: selection of the process to be assessed, formation of a multidisciplinary team, description and mapping of the process, identification of potential failure modes and their causes and effects, assessment of severity, occurrence and detectability, calculation of the risk priority number (RPN) and identification of corrective actions. These steps are described below.

During the 6‐month testing period, a total of 19 clinical issues were proposed as potential topics for FMEA. Of these, 8 risk assessments were completed. The remaining 11 proposed processes were not assessed: in 7 cases, the assessment could not be conducted due to lack of time among the participants; in 3 cases, the responsible person did not consider the FMEA necessary and in 1 unit, there was interest in conducting the FMEA assessment, but no suitable process was identified. The person who decided on whether the risk assessment should be carried out was always in charge or responsible for the unit or the process. Among the eight completed FMEA assessments, the processes were selected based on reported adverse events (*n* = 1), the initiation of a new process (*n* = 4), or recent changes made to an existing process (*n* = 3). For example, there was a need for a risk assessment in the paediatric ICU because a new project had been started to replace the electronic health record.

According to the FMEA process, the team members for the risk assessment were selected based on their profession, experience and knowledge of the specific topic. The aim was to include participants representing different areas of the process [31]. Participants were invited by the person who decided on the assessment of the process and participation was voluntary. The selection of participants was based on their familiarity with and expertise in the processes. The number of team members in each assessment varied between 3 and 6 (mean = 4). The team leader (A.F‐H) took part in every assessment meeting. Team leader facilitated the meetings, introduced the FMEA method, guided the discussion and documented the results

The teams (*n* = 7) met twice with one team only meeting once. The meetings lasted from 2 to 4 h. During one risk assessment, as an exception, the members agreed to hold the second meeting online due to difficulties in finding a suitable time for a personal meeting. When describing the processes under assessment, teams used either verbal descriptions (*n* = 4) or process flow charts (*n* = 4). Verbal descriptions were summarised by the team leader (A.F‐H). Each meeting proceeded according to FMEA process. After the process description, the multidisciplinary teams identified failure modes (FMs) for every process step. For each FM potential effects and causes were analysed from different perspectives (patient, employee, organisation) and current controls were reviewed. Values for severity, occurrence and detection were determined through structured group discussion, and consensus was reached among all team members. No individual scoring was used. Consensus was required for each rating before proceeding to RPN calculation. Brainstorming was used to support the process, allowing representatives from different professional groups to contribute with their own perspectives and expertise. The team leader documented the data. A Microsoft Excel based FMEA template, developed by Lean Six Sigma, was used for data collection.

In this study, scales for severity (S), occurrence (O) and detection (D) were revised [28]. The revision aimed to clarify the scales which were intended to be consistent with those currently in use in the patient safety risk assessment programme in the department. The original content and purpose of the scales were preserved throughout the revision process and the revised scales were subsequently applied in the present study. The scales for S and O ranged from 1 to 5, with higher scores indicating more severe consequences and more frequent occurrence, respectively, while the scale for D ranged from 1 to 4, with higher scores indicating lower detectability. The risk priority numbers (RPNs) were automatically calculated *(S × O × D* = *RPN)*. The RPN could vary from 1 to 100. The FMEA template and modified scales were tested in a risk assessment before the beginning of the study. No issues arose during the testing phase.

For every possible failure mode, recommended actions were considered. The responsible persons for corrective actions were named. If the person responsible was outside of the team in question, she or he was not named before consultation.

### 2.4 Ethical Consideration

Ethical approval was secured from the Ethics Committee for Human Sciences at the University of Turku, Health Care Division. Consent for the study was obtained from the Wellbeing County of Southwest Finland. Participation in the study was voluntary for all individuals.

## 3 Results

In the study, totally eight processes were assessed with FMEA. Because of the aim of the study, no limits were set on the nature of the process. Processes were divided into four application areas [20]: healthcare processes (*n* = 3), hospital management (*n* = 1), hospital informatisation (*n* = 2) and medical equipment or production (*n* = 2). Almost all the teams (*n* = 7) met twice. Each meeting lasted an average of 2 h (Table 1). The results are represented according to the application areas.

**Table 1:** Risk assessment processes in the paediatric department.

	Name of the risk assessment process	Unit	Number of team members	Time spent on risk assessment (h)	Number of meetings	Number of failure modes (FMs)	Profession of team members
Healthcare process	Botox process	Paediatric day hospital	5	3.5	2	11	Physiotherapist (2), nurse, assistant nurse manager, team leader
	Brain injury process	Paediatric neurological unit	6	3	2	6	Paediatrician, resident physician, nurse, psychologist, occupational therapist, team leader
	Change in the operation of the neurology outpatient clinic	Paediatric neurological unit	4	4	2	7	Nurse (2), rehabilitation counsellor, team leader
Hospital management	The change in the role of assistant nurse manager	Paediatric intensive care and surgical units	4	2.5	2	6	Assistant nurse manager (2), nurse manager, team leader
Hospital informatization	Implementation of a digital patient application	Paediatric department	4	3	2	11	Paediatrician, clinical nurse specialist, assistant nurse manager, team leader
	Implementation of a new electronic patient record system	Paediatric intensive care unit	3	3	2	7	Clinical nurse specialist, nurse manager, team leader
Medical equipment or production	The use of glass bottles in breastmilk pasteurisation	Neonatal intensive care unit	3	2	1	5	Practical nurse, assistant nurse manager, team leader
	The use of smart pumps	Paediatric unit	4	3	2	6	Assistant nurse manager (2), nurse manager, team leader

### 3.1 Healthcare Processes

The three assessments dealt with healthcare processes, including the Botox‐process, the brain injury process, and the operations of neurology outpatient clinic. In the assessment of the Botox‐process, there were employees from four different professions. The team assessing the brain injury process consisted of six different professional groups. This group was the most multidisciplinary, probably because there are various professionals working in this process. In the assessment of the neurology outpatient clinic, the team members were from three different professional groups. In the healthcare processes assessments, teams found from 6 to 11 different failure modes (FMs). RPN varied from 2 to 27.

### 3.2 Hospital Management

One risk assessment was categorised in hospital management. Assessment of the change in the assistant nurse manager role was conducted to evaluate the risks associated with the adjustment and to respond to them in advance. Six FMs were found and the RPN range was 4–24. Team members were nurse managers or assistant nurse managers from the unit.

### 3.3 Hospital Informatization

Two assessments were classified into hospital informatisation. The implementation of a new electronic patient record system in the paediatric ICU and the implementation of a digital patient application involved the introduction of either new or unfamiliar technological applications. In these assessments, teams found altogether 18 failure modes. The RPN varied from 3 to 36. The results of the assessment of a digital patient application were later used in the project meetings to ensure the safety of the implementation.

### 3.4 Medical Equipment or Production

Two assessments were categorised into medical equipment or production, including the process of using glass bottles in breastmilk pasteurisation, and the use of smart pumps. For the former, the need for an assessment arose from patient safety reports. The latter related to equipment. The team (*n* = 3) met once and completed the assessment which might be due to the compact nature of the process. During these 2 assessments the teams found 11 FMs. The RPN varied between 1 and 24.

In this study, the number of all FMs (*n* = 59, *M* = 6.75 FMs/process) was low compared to previous studies [33, 36, 45]. The RPN varied from 1 to 36. The value of RPN is used to prioritise corrective actions. A low RPN does not require immediate corrective actions, whereas a high‐risk RPN indicates that corrective actions should be taken without delay. Due to the relatively small number of FMs, the teams were able to develop corrective actions for most of them (*n* = 52).

### 3.5 Corrective Actions

The teams prioritised failure modes with high RPN values in designing corrective actions as their primary task was to develop appropriate measures for these FMs. In the study, altogether 52 corrective actions were planned for 8 processes, *M* = 6.5 corrective actions per process (Table 2). For FMs that already had existing effective controls (*n* = 4), no actions were planned. For example, in the Botox‐process one failure mode was ‘difficulties in applying a cast to the patient’. An illustrated manual and new instructions for casting had just been implemented so no corrective actions were planned. In the breastmilk pasteurisation process it was decided to replace the current glass bottles with another type of bottles as a corrective action. With this, the team aimed to improve four out of all five failure modes. Implementation of the corrective actions is not reported in this study.

**Table 2:** Severity, occurrence, detectability and corrective actions.

	Name of the process	Severity (S) 1–5	Occurrencce (O) 1–5	Detectability (D) 1–4	Risk priority number (RPN)	Range	Examples of corrective actions
Healthcare processes	Botox process 7 FM	3	3	2	18	4–18	*Implementation of a check‐list for patients’ Botox treatment days*
		3	2	1	6		
		3	1	3	9		
		3	2	1	6		
		2	2	2	8		
		2	2	1	4		
		2	2	2	8		
	Brain injury process 6 FM	3	2	2	12	8–27	*Setting up a new digital waiting list for nurses’ follow‐up patients*
		2	2	2	8		
		3	3	3	27		
		2	2	2	8		
		4	2	2	16		
		2	2	2	8		
	Change in the operation of the neurology outpatient clinic 7 FM	3	2	1	6	2–9	*Pre‐call to a patient before first appointment at a new location*
		3	1	1	3		
		3	2	1	6		
		2	3	1	6		
		3	1	3	9		
		2	1	1	2		
		2	1	1	2		
Hospital management	The change in the role of the assistant head nurse 6 FM	2	4	2	16	4–24	*Strentghening the role of the physician in charge of the unit in communication and operational organization*
		3	4	2	24		
		2	3	2	12		
		3	3	1	9		
		3	3	1	9		
		2	2	1	4		
Hospital informatization	Implementation of a new electronic patient record system 7 FM	3	4	1	12	12–36	*During the implementation phase, each shift must have a key user or superuser present*
		2	4	2	16		
		3	3	2	18		
		2	3	2	12		
		2	4	3	36		
		2	4	2	16		
		2	3	2	12		
	Implementation of a digital patient application 9 FM	1	3	1	3	3–36	*The aim is to modify the application so that incoming messages will be visible to all employees*
		2	3	2	12		
		4	3	3	36		
		4	4	2	32		
		3	3	2	18		
		2	2	1	4		
		2	3	3	18		
		2	3	2	12		
		3	2	2	12		
Medical equipment or production	The use of glass bottles in breast milk pasteurisation 5 FM	3	2	2	12	1–16	*The weekly meetings will be upheld, but in urgent situations, they can be conducted remotely*
		1	1	1	1		
		2	4	2	16		
		1	2	1	2		
		2	3	1	6		
	The use of smart pumps 6 FM	2	2	2	8	8–24	*Wi‐Fi‐enabled infusion pumps are used so that device alerts are sent directly to nurses*
		3	2	2	12		
		2	2	2	8		
		3	4	2	24		
		2	4	2	16		
		2	3	2	12		

Abbreviation: FM, Failure Modes.

Some corrective actions were simple and were considered not to require much effort in implementation. For example, informing other professionals about the changed care process (change in the operation of the neurology outpatient clinic). In contrast, teams invented corrective actions that encompassed multiple aspects and were complex in nature. For example, the brain injury process team wanted to publish care instructions online and to organise training on brain injuries for students and professionals.

## 4 Discussion

The aim of the study was to test and introduce the Failure Mode and Effect Analysis (FMEA) method into various processes in a paediatric and adolescent hospital department by applying action research methodology. The combination of action research and FMEA proved to be a successful approach. To the best of our knowledge, this is the first study where FMEA has been tested within this scope of paediatric and adolescent care in a hospital setting. Action research and the FMEA procedure bear resemblance as both enable cyclic processes over time [31, 42], both have been used in implementations [41] and require strong collaboration with practitioners [20, 38].

Action research has been used to improve care processes [39] and patient safety [40]. In our study, the insider role of the researcher (team leader) supported the aim. Firstly, from the researcher's point of view the familiarity with the department, staff and processes was useful. Secondly, the teams benefited from the team leader's insight in paediatrics. The insider role also facilitated the assessment processes from the planning to the production of results. Action research is an appropriate method to improve the quality and relevance of paediatric research [41]. Based on our experience, it is a suitable method for supporting quality improvement of specific processes in paediatric hospital care.

Although team sizes were smaller than in some earlier studies [20, 24, 26, 45], the assessments were successfully completed and the smaller teams may have facilitated more focused discussion. The lack of professional participants’ time made it difficult to assemble larger groups. For the same reason, seven assessments were not carried out in this study.

FMEA is based on expert judgement and the use of numerical scoring for severity, occurrence and detection, which may introduce variability and uncertainty into risk prioritisation. To address this limitation, fuzzy FMEA approaches have been proposed in the literature to better capture uncertainty in expert assessments and to strengthen the robustness of risk evaluation [46, 47]. In the present study, a conventional FMEA approach was used to ensure feasibility and ease of implementation in a real‐world clinical setting. This approach supported practical risk assessment within routine clinical work without adding methodological complexity. Future research could explore fuzzy FMEA methods to further develop risk prioritisation approaches in healthcare environments.

All application areas of FMEA [20], were identified in this study, with healthcare processes being the most common. Adverse events in paediatrics hospital care are generally related to incidents such as medication errors [8, 13, 14] and to the use of medical devices [8]. The use of smart pumps represents a process that is closely associated with these risk factors. In this study, only a subprocess of smart pump use was assessed. Its successful application is consistent with previous findings suggesting that FMEA can be applied to specific parts of a process, provided that the process is clearly defined at the outset [29]. The study suggests that FMEA may be a suitable method for less typical processes, such as the use of glass bottles in breast milk pasteurisation process. This is an important consideration, given the specific characteristics of paediatric and adolescent healthcare.

This study is beneficial for the department. During the study, the team members had an opportunity to become familiarised with the FMEA method. Study also promoted interprofessional collaboration. Implementation of corrective actions and their effects became available to team members and the department for future use in order to prevent AEs and support patient safety. The study provided a tested model for systematic risk assessment that can be applied in future process development and implementation projects.

To our knowledge, very little research has been done on the opinions of the team members. Future studies should consider focusing on this aspect. There is also a lack of studies reporting the follow‐up of corrective actions [20]. For future research, it would be interesting to investigate the effects of FMEA at a wider level, for example on a departmental level. This could for instance be accomplished by measuring the patient safety culture. Based on the present results, we encourage the use of the method in implementation and development of clinical practice in paediatric and adolescent hospital care.

### 4.1 Strength and Limitations

The study used an action research approach in order to test FMEA in a paediatric department. The method ensured that the teams were guided through the process assessment with the team leader's support, taking the experience and needs of the participants into consideration. It is possible, that the researcher's role as the FMEA team leader might have had some effects for example on the teamwork, because of the dual role as a researcher and a member of the department staff.

Eight assessed processes can be considered as a good result in view of the time‐specific requirements of FMEA [30] in combination with the commonly recognised challenges of staffing shortages [29]. The time allocated to the assessment was not restricted. However, the teams spent less time on the assessments compared to previous studies [33, 34]. The objective was to form multidisciplinary teams to ensure reflection among participants. The quality of the assessments might have been further improved by involving even more diverse teams representing a broader range of professional groups. There are also recommendations that support more than five team members to enhance the effectiveness of FMEA [20]. These factors may be regarded as limitations of the study. Conversely, from the testing perspective, the number of participants did not complicate the testing. The participants’ contributions in the phases of analysis and drawing conclusions were limited, as their contributions in the study mainly took place in the phases of planning and acting. The validity of the study could have been improved by the members to assess the results, which was not included the study. However, the participants were asked to provide feedback after the assessments, and the perspectives were used as data in order to draw conclusions.

The empirical results were gained from a paediatric and adolescent hospital and are therefore not necessarily transferable other health care setting.

## 5 Conclusions

The Failure Mode and Effect Analysis is a suitable method for assessing various processes in a paediatric and adolescent hospital. Action research was considered to be an appropriate method to support the testing of FMEA and to introduce a proactive risk management method for health care. The results are promising but are as such not necessarily transferable to other health care settings.

## Funding

The authors have nothing to report.

## Conflicts of Interest

The authors declare no conflicts of interest.

## Data Availability

The data that supports the findings of this study are available from the corresponding author upon reasonable request. No permission was required to reproduce material from other sources.
